# The relationship between work requirements and mental distress in hospital staff: the chain mediating effects of rumination and work recovery classes

**DOI:** 10.1186/s40359-025-02588-1

**Published:** 2025-03-17

**Authors:** Jinjin Li, Xinxin Ma, Wenhao Pan, Huihui Ke, Zhenghua Xiao

**Affiliations:** 1 School of Psychology, Guizhou Normal University, Guiyang, 550025 China; 2 School of Public Administration, South China University of Technology, Guangzhou, 510641 China; 3 Guizhou University of Finance and Economics, Guiyang, 550025 China; 4 The Second Affiliated Hospital of Guizhou, University of Traditional Chinese Medicine, Guizhou, China

**Keywords:** Hospital staff, WR, Latent profile analysis, Chain mediated modelling

## Abstract

**Background:**

Heavy work requirements can lead to significant increases in depression anxiety and stress among hospital staff. However, there is limited research considering the role of work recovery (WR) and rumination in this relationship, particularly how poor WR can create a vicious cycle of negative psychological outcomes for medical staff.

**Objective:**

In this cross-sectional observational study, the aim is to explore high-risk WR classes among hospital staff. By constructing chain mediation models according to the WR classes, the study seeks to identify which class of medical staff is most affected by work requirements in terms of mental distress, and to examine the chain mediation effects of rumination and WR classes.

**Methods:**

The cross-sectional observational study utilized Latent Profile Analysis (LPA) and Structural Equation Modeling (SEM) to investigate the relationships among various constructs. It employed the Work Requirements Scale (WRS), the Recovery - Stress Questionnaire (RESTQ), the Depression Anxiety Stress Scales (DASS), and the Revised Emotion Control Questionnaire (RECQ) to survey a sample of 889 hospital staff at a tertiary hospital.

**Results:**

The findings revealed three distinct WR classes among hospital staff. Significant associations were found between work requirements and mental distress among medical staff in the low and moderate WR classes. Additionally, in these two classes, there were significant relationships in which rumination and WR classes had a chain-mediated nature about work requirements and mental distress. This suggests that future intervention studies should focus on these two classes and develop psychological health interventions for hospital staff according to different WR classes.

**Supplementary Information:**

The online version contains supplementary material available at 10.1186/s40359-025-02588-1.

## Introduction

Hospital staff encounter a myriad of challenges that adversely affect their mental health compared with other professions. Researchers have conducted a comparative study between hospital nurses and teachers [1], and the results show that hospital nurses have significantly higher levels of anxiety and depression than the teacher group due to factors such as the complexity of the work environment, irregular working hours, and the pressure of facing patients’ life and death; In addition, studies have compared the occupational burnout and work-life balance satisfaction between doctors and the general working population, and found that the mental health problems of doctors are more prominent [2]. The increasing number of patients, combined with concerns about occupational safety and challenging work environments, has significantly elevated the levels of stress, anxiety, and depression among hospital staff [3, 4]. Research indicates that the demands of the healthcare profession profoundly affect the mental health of hospital staff, particularly those in direct patient care roles, who often experience higher rates of mental health disorders due to chronic work-related stress [5]. The pressure to manage complex cases and achieve positive patient outcomes further exacerbates the risk of adverse psychological effects, including anxiety and depression [6]. These mental health issues not only jeopardize the well-being of hospital staff but also compromise the quality of patient care, creating a vicious cycle of increased stress and emotional distress. According to relevant research [7], “increased stress” usually refers to a physical and psychological response that occurs when faced with external or psychological stressors, such as physiological changes such as increased heart rate and blood pressure, as well as psychological feelings such as anxiety and worry. And ‘emotional distress’ is a deeper emotional state, which is an unpleasant feeling experienced when unable to adapt to stressors. It affects emotions and thinking and may also affect behavior [8]. Long term exposure to high levels of stress may lead to emotional distress, manifested as persistent anxiety, depression, helplessness, etc [9]. As healthcare systems continue to evolve, it is crucial to foster a supportive and sustainable work environment to address the mental health issues faced by hospital staff.

Hospital staff face significant psychological challenges in effectively treating patients and managing their daily work [10]. These hospital staff, who are directly in contact with patients, are exposed to stressful and unpredictable work environments, including long and uncertain working hours, high work requirements, vague roles, and exposure to human suffering [11]. Nevertheless, the work requirements and job pressures experienced by hospital staff are often taken for granted or viewed as a problem for the future. Therefore, the excessive work requirements are likely to cause additional mental health problems for medical staff. For example, previous studies have shown that increased work requirements and job pressures can lead to higher rates of mental distress among hospital staff [12]. Specifically, depression prevalence among hospital staff was 22.8%, stress ranged from 16.5 to 48.3% [12, 13], and anxiety increased to between 22.6% and 36.3% [14]. However, most studies focus on the impact of work requirements on hospital staff, and the specific mechanisms that influence mental distress remain unclear. Studies have shown that the workload dimension in job requirements is positively correlated with psychological stress [15], but the underlying mechanisms of psychological adjustment and cognitive processing differences in why individuals experience varying degrees of depression and anxiety when faced with the same workload are not fully understood. Although research has confirmed the association between job demands and psychological stress [16], difficulties have been encountered in exploring the underlying mechanisms that specifically affect mental health. The study suggests that individual personality traits play a complex regulatory role, but its exact action pathway has not been elucidated.

Rumination can be defined as passively and repetitively focusing on one’s symptoms of distress and the circumstances surrounding these symptoms [17]. Research has established that negative rumination is significantly associated with increased levels of mental distress [18, 19]. Conversely, work recovery (WR) is the process through which individuals unwind from job pressures, facilitating both psychological and physical restoration [20, 21]. The effectiveness of this recovery process is largely determined by an individual’s ability to disengage (or disconnect) from work-related thoughts [22]. Impaired work recovery can have detrimental effects on an individual’s health. Notably, rumination has been identified as a factor that contributes to impaired work recovery [23]. Additionally, existing evidence positions rumination as a significant mediator in the relationship between work requirements and the development of mental distress [22, 24, 25]. Work requirements are a source of occupational stress for hospital employees, and excessively high work demands can make employees passively and repeatedly focus on their mental suffering and related situations [26]. Ruminating thinking is closely related to psychological pain symptoms such as depression, anxiety, and stress [27, 28], which can increase the degree of psychological pain for employees. Hospital employees, facing a large number of patients, complex medical conditions, and high-intensity work, are prone to repeatedly thinking about work pressure, which can lead to negative psychological states. Previous studies have shown that when employees have a high level of work recovery, they can effectively relieve work stress and achieve physical and mental recovery [29, 30]. This good state of recovery helps to weaken the production of rumination, and even when faced with high job demands, it can reduce the psychological pain caused by rumination. On the contrary, if employees have a low level of work recovery and cannot effectively relieve work pressure, under high work requirements, rumination is more likely to occur and difficult to interrupt, which will exacerbate psychological pain. Despite this understanding, it remains unclear whether work recovery itself has a mediating effect on the relationship between work requirements and mental health outcomes, e.g., depression, anxiety, and stress. Further research is needed to explore this potential mediating pathway.

Research has increasingly recognized the significant role of WR in the relationship between work requirements and mental health outcomes, such as depression, anxiety, and stress [31, 32]. WR is a short-term reaction to the exertion of energy in response to work requirements and may not necessarily indicate a problem. However, when hospital staff cannot effectively engage in recovery strategies, the negative consequences of excessive work requirements, compounded by work-related rumination, can accumulate [23]. This accumulation can ultimately lead to more severe physical and mental health issues, including depression, anxiety, and stress [23]. Therefore, monitoring WR is crucial as it serves as an early indicator of these mental health issues [33]. By identifying hospital staff who are at risk of accumulating job strain and pressure, intervention strategies can be implemented before impaired WR progresses into more serious health problems. Developing a good ability to engage in recovery strategies may allow hospital staff to reduce job pressure and restore energy [34, 35]. Conversely, inadequate recovery can have cumulative adverse effects, potentially leading to heightened depression, anxiety, and stress. In other words, even when recovery opportunities are available, hospital staff may struggle to return to optimal health due to impaired recovery mechanisms [36]. Those experiencing diminished WR may feel an urgent need for breaks, which can lead to a temporary resistance to fulfilling work requirements or taking on additional responsibilities [37–39]. This situation aligns with the concept of the ‘Recovery Paradox’ [22], where excessive work requirements may amplify depression, anxiety, and stress through increased rumination and compromised WR. This dynamic can create a vicious cycle, particularly among individuals exhibiting low WR.

Existing research predominantly employs questionnaires to assess WR, and a lower score indicates that hospital staff had a worse WR level [40]. However, recent attention has focused on the interrelated and multifaceted occurrence of different types of WR rather than in isolation [41]. A recent study utilized Latent Class Analysis (LCA) to classify work requirements and examine result differences across WR classes [41]. LCA studies offer advantages, such as identifying distinct subclasses within a population that traditional methods may overlook [42]. This nuanced understanding enables tailored interventions for each subclass and enhances insights into variable interactions affecting WR patterns. Furthermore, LCA facilitates the examination of heterogeneity within a sample, which can lead to more effective resource allocation and targeted support strategies. Overall, LCA is a valuable tool for developing intervention strategies that address the diverse characteristics of WR patterns. For example, Sun et al. (2024) uniquely used LCA to identify four patterns of work recovery need among 243 construction staff, finding that those with high work recovery need exhibited significantly greater rumination, work pressure, and depressive symptoms than those with a low work recovery need [41].

Despite WR’s importance, a significant literature gap exists. Research indicates that recovery needs may vary by occupational and individual characteristics [41]. Identifying WR’s multifaceted aspects is essential for developing effective interventions for high-risk hospital staff. Understanding WR patterns and their links to work requirements, rumination, and mental health is crucial for designing targeted interventions to enhance health and support among specific hospital staff groups. This research aims to use LCA to investigate WR patterns in hospital staff and explore their relationships with work requirements, rumination, and mental health. Specifically, this study has two primary aims: (1) to identify individual differences in WR patterns among hospital staff, and (2) to assess whether chain mediation models involving rumination and WR apply across hospital classes with varying WR patterns in linking work requirements to mental distress. For the second aim, the specific hypotheses are: (a) classes with distinct WR patterns will differ significantly in work requirements, rumination, WR, and mental health indicators such as mental distress; and (b) rumination and WR will sequentially mediate the relationship between work requirements and mental distress for staff with impaired WR, but not for those with adequate WR. In previous studies, although some literature focused on the impact of job requirements on the mental health of hospital staff, there were certain limitations in the depth and breadth of research. Most studies only focus on the direct correlation between job demands and psychological symptoms such as depression, anxiety, and stress [12], with little exploration of the underlying mechanisms. For example, although studies have shown that job requirements increase the psychological burden on hospital staff [14], the mediating role of rumination and work recovery in this process, especially the chain mediation effect, has not been fully studied.

Based on the above analysis, the innovation of this study lies in the following aspects: Firstly, for the first time, a chain mediation model was constructed among medical staff based on job recovery categories, comprehensively exploring the complex relationship between job requirements, rumination, job recovery, and mental distress, providing a new perspective for understanding the mental health issues of hospital staff. Secondly, the use of LPA to classify work recovery breaks through the limitations of traditional single evaluation methods, allowing for a more detailed characterization of the heterogeneity of hospital staff work recovery and providing a more scientific basis for targeted interventions in the future. Finally, the research results identified high-risk groups under different categories of work recovery, providing empirical support for the development of differentiated mental health interventions and helping to improve the accuracy and effectiveness of interventions, which has not been fully reflected in previous studies.

## Methods

### Data collection and participants

This study belongs to cross-sectional observational research. 889 healthcare workers will be tested through the “Wenjuanxing” online assessment system in March and April 2023. Considering the dispersed work locations and inconsistent working hours of hospital employees, online questionnaires can more efficiently cover a wider sample group, ensuring that employees from different departments and positions have the opportunity to participate in the survey. We used a professional online survey platform to ensure the stability of the questionnaire and the accuracy of data collection. Before distributing the questionnaire, we conducted multiple tests and optimizations on its content and format to improve its quality and comprehensibility. At the same time, in order to ensure the response rate and validity of the questionnaire, we provided a detailed explanation of the research purpose and the importance of participation at the beginning of the questionnaire, and promised to strictly keep the information of the participants confidential. During the questionnaire distribution process, we sent the questionnaire link to employees through internal work groups, email, and other channels within the hospital, and arranged for a dedicated person to follow up on the questionnaire collection status, providing appropriate reminders to employees who did not fill out the questionnaire in a timely manner. This study has been approved by the Ethics Committee of the Second Affiliated Hospital of Guizhou University of Traditional Chinese Medicine, with approval number EC202003, and all participants have provided informed consent.

A total of 889 hospital staff from a tertiary hospital in Guizhou Province were assessed using a convenience sampling approach. After excluding duplicate responses and those with response times beyond three standard deviations, 847 valid questionnaires were obtained; this means that 42 of the initially collected data were excluded, accounting for 4.73% of the total collected data, yielding an effective response rate of 95.27%. The sample comprised 259 males (30.6%) and 588 females (69.4%), with a mean age of 34.45 years (SD = 7.08 years). We consider this 4.73% of data as broadly defined ‘missing data’ (data that was not included in the analysis due to data quality issues), but these data are not traditionally missing due to reasons such as participants not answering questions. In the actual analysis process, due to the requirement for data integrity in the statistical methods we used, and the fact that the excluded data were mainly due to abnormal answers rather than random missing data, we did not specifically fill in these data.

### Measurement tools

*Work Requirements* were assessed using the Work Requirements Scale (WRS) [43]. The Chinese version of WRS has been applied among the medical staff in China [43]. The scale consists of 15 items, including subscales for professional knowledge requirements, work overload, role ambiguity, emotional demands, role conflict, and work-family conflict. For example, in order to complete work, I often have to put in extra effort; I have so much work to do that I have to give up my hobbies. The scale employs a 5-point scoring system, ranging from 1 (strongly disagree) to 5 (strongly agree). A higher score on this scale suggests that individuals need to meet higher work requirements and standards. In this study, the Cronbach’s α coefficient is 0.89. The results of confirmatory factor analysis showed that the model fit well (CFI = 0.944; TLI = 0.917; SRMR = 0.06; RMSEA = 0.07).

*Work Recovery* (WR) was assessed using the Recovery-Stress Questionnaire (RESTQ) [44]. The RESTQ has been applied in the healthcare workforce [45]. The questionnaire consists of 18 items, including subscales for physiological recovery, social recovery, sleep quality, and sense of achievement. The scale employs a 7-point scoring system, ranging from 0 (never) to 6 (always). Example of items: I slept well, relaxed, and visited some close friends. Higher scores on the RESTQ indicate better post-WR for the individual. In this study, the Cronbach’s α coefficient is 0.93. Confirmatory factor analysis was conducted to investigate the structure of the RESTQ, and the results showed that the model fit well (CFI = 0.922; TLI = 0.907; SRMR = 0.053; RMSEA = 0.076).

*Mental Distress* was assessed using the Depression Anxiety Stress Scales (DASS) [46]. The Chinese version of DASS has been applied among medical staff in China [47]. The scale comprises 21 items, including three subscales for anxiety, depression, and stress. For example, I find it difficult to calm myself down; I can’t be optimistic at all. The scale employs a 4-point scoring system, ranging from 0 (strongly disagree) to 3(always strongly agree). Higher scores on the DASS indicate more severe levels of depression, anxiety, or stress. In this study, the Cronbach’s α coefficient is 0.92. The results of confirmatory factor analysis showed that the model fit well (CFI = 0.906; TLI = 0.90; SRMR = 0.045; RMSEA = 0.059).

*Rumination* was assessed using the rehearsal subscale of the Revised Emotion Control Questionnaire (RECQ) [48]. The scale was revised by Chinese scholars, including subscales for rehearsal, emotional inhibition, aggression control, and benign control [49]. The scale employs a 2-point scoring system, ranging from 1 (no) to 2(yes). In this study, only the rehearsal subscale was utilized; this scale contains 8 items. Higher scores reflect a higher tendency to ruminate on stressful or distressing events; for example, even after a long time has passed, I still can’t forget those things that once annoyed or angered me. In this study, the Cronbach’s α coefficient is 0.75. The results of confirmatory factor analysis showed that the model fit well (CFI = 0.941; TLI = 0.918; SRMR = 0.042; RMSEA = 0.075).

### Data analysis

Data were entered by two persons using EpiData 3.1 software for cross-validation. Harman’s single-factor test was used to assess common method bias [50]. The results showed that 14 factors with feature roots greater than 1 were extracted, and the variance explained by the first factor was 25.529%, which is less than 40%. This suggests that there is no severe common method bias. First, Latent Profile Analysis (LPA) was performed on the WR of hospital staff using Mplus 7.4, with suitable model indices employed to identify the optimal classification. Subsequently, according to the WR classes identified by LPA, SPSS 22.0 was employed to perform descriptive statistics and correlation analysis on demographic variables and recovery-related variables such as rumination, work requirements, and mental distress. Finally, based on the latent profile classification results of WR, SPSS Process 4.1 was employed to construct structural equation models, assessing the chained mediation effects of rumination and WR between work requirements and mental distress for each hospital staff class.

Using Pearson correlation coefficient to determine the correlation between variables, according to Cohen’s suggestion [51], *r* = 0.1–0.3 indicates weak correlation, *r* = 0.3–0.5 indicates moderate correlation, and *r* = 0.5-1.0 indicates strong correlation. The evaluation indices for the LPA model include the Akaike Information Criterion (AIC), Bayesian Information Criterion (BIC), Adjusted BIC (aBIC), Entropy, Lo-Mendell-Rubin (LMR) likelihood ratio test, and Bootstrapped Likelihood Ratio Test (BLRT). Among them, AIC, BIC, and aBIC are used to compare models, with lower values indicating better fit. Entropy, ranging from 0 to 1, reflects classification accuracy, where values above 0.80 suggest over 90% misclassification. Significant LMR and BLRT values (*p* < 0.05) indicate model K explains more variance than model K-1 [52].

## Results

### The latent class of WR

LPA model fit indices were evaluated for solutions with 2 to 5 latent classes to determine the best-fitting model that captures distinct patterns of WR (Table 1). Using the four WR scale dimensions as indicators, LPA revealed that the 3-class and 4-class models, with Entropy values over 0.8, fit better than the 2-class and 5-class solutions. The AIC, BIC and aBIC values gradually decreased as the number of classes increased. The 4-class model shows lower AIC, BIC, and aBIC values and higher Entropy than the 3-class model. However, its LMR(*p*) exceeds 0.05, with less pronounced decreases in AIC, BIC, and aBIC. Considering the model fit indices, the 3-class solution was the most appropriate.

**Fig. 1:**
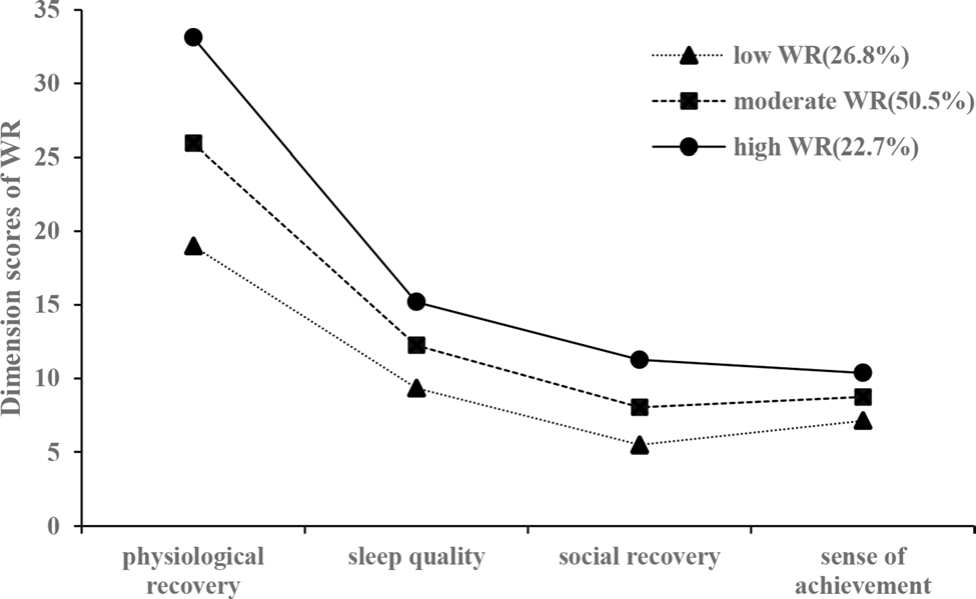
Probabilities of WR for Three Latent Classes

The mean dimension scores for each class are illustrated in Fig. 1. Hospital staff in Class 1 (26.800%) were characterized by having the lowest scores on all dimensions and were labelled the “low WR class” [physiological recovery: 18.740 ± 3.026, sleep quality: 9.322 ± 2.745, social recovery: 5.405 ± 1.474, sense of achievement: 7.062 ± 1.821)]. Hospital staff in Class 3 (22.700%) had the highest scores on all dimensions and were defined as the “high WR class” [Same as above: 33.229 ± 3.122, 15.229 ± 2.635, 11.359 ± 1.444, 10.396 ± 1.870]. Class 2 (50.500%) was named the “moderate WR class” [Same as above: 25.986 ± 2.836, 12.222 ± 2.526, 8.021 ± 1.582, 8.743 ± 1.637], as the mean dimension scores were higher than those in Class 1 but lower than those in Class 3.

**Table 1:** Goodness-of-Fit indices for WR class

Model	K	AIC	BIC	aBIC	Entropy	LMR(*p*)	BLRT(*p*)	Latent Class Probability
1-class	8	17530.304	17568.238	17542.832	-	-	-	1
2-class	13	16672.438	16734.080	16692.796	0.756	< 0.001	< 0.001	0.589/0.411
3-class	18	16333.971	16419.321	16362.159	0.804	< 0.001	< 0.001	0.268/0.505/ 0.227
4-class	23	16283.595	16392.654	16319.613	0.849	= 0.146	< 0.001	0.494/0.226/0.273/0.008
5-class	28	16227.524	16360.292	16271.373	0.789	= 0.587	< 0.001	0.219/0.427/0.129/0.007/0.217

Note: K is the number of freely estimated parameters

### Descriptive and correlation statistics based on WR classes

The study examined demographic and related variable differences among hospital staff across WR classes (Table 2). Specifically, the three classes showed no significant differences in education (χ^2^(8) = 17.156, *p* = 0.067) and gender (*χ*^2^(2) = 10.344, *p* < 0.111). Significant differences were observed among the classes in professional titles (*χ*²(8) = 23.098, *p* = 0.003), with a higher proportion of staff lacking titles in the high WR class compared to the low and moderate classes. Job position distributions also differed significantly (*χ*²(8) = 38.868, *p* < 0.001), with more medical and nursing staff in the low and moderate WR classes. Marital status (*χ*²(6) = 17.527, *p* = 0.004) and fertility (*χ*²(2) = 11.508, *p* = 0.003) distributions showed higher proportions of married staff and those with children in the low and moderate classes. In contrast, no significant differences were found in working years [*F*(2,844) = 0.290, *p* = 0.748, *η*²=0.001] and age [*F*(2,844) = 0.754, *p* = 0.471, *η*²=0.002].

The study investigated class differences in work requirements, rumination, WR, and mental distress. Significant differences were found in self-reported work requirements [*F*(2,844) = 72.152, *η*²=0.146, *p* < 0.001], with the low and moderate WR classes reporting higher requirements than the high WR class. Rumination also varied significantly [*F*(2,844) = 64.590, *η*²=0.133, *p* < 0.001], being more pronounced in the low and moderate classes. WR differences were substantial [*F*(2,844) = 1965.660, *η*²=0.823, *p* < 0.001], with lower WR reported in the low and moderate classes. Additionally, mental distress levels differed significantly [*F*(2,844) = 173.871, *η*²=0.292, *p* < 0.001], with higher levels in the moderate and high WR classes compared to the low WR class. The hypothesis proposed earlier has been validated, which states that “classes with distinct WR patterns will differ significantly in work requirements, rumination, WR, and mental health indicators such as mental distress”.

**Table 2:** Sample characteristics by WR classes

	1. Low WR (26.8%)	2. Moderate WR (50.5%)	3. High WR (22.7%)	Difference Tests	Post-hoc Analysis
**Gender(N(%))**				χ^2^(2) = 0.72	
Male	70(8.26)	126(14.88)	63(7.44)		-
Female	157(18.54)	302(35.65)	129(15.23)		-
**Have children (N(%))**				χ^2^(2) = 11.51, *p* = 0.003^**^	
Yes	177(20.89)	292(34.47)	122(14.40)		1 < 2, 1 > 3
No	50(5.90)	136(16.06)	70(8.26)		-
**Marital status (N(%))**				χ^2^(6) = 17.53, *p* = 0.004^**^	
Married	189(22.31)	333(39.32)	132(15.58)		1 = 2 > 3
Widowed	0(0)	1(0.1)	1(0.1)		-
Divorced	9(1.06)	9(1.06)	8(0.94)		-
Unmarried	29(3.42)	85(10.04)	51(6.02)		-
**Education (N(%))**				χ^2^(8) = 17.16	
High school or below	19(2.24)	43(5.08)	24(2.83)		-
Associate degree	133(15.7)	258(30.46)	117(13.81)		-
Undergraduate degree	65(7.68)	102(12.04)	38(4.49)		-
Graduate degree	10(1.18)	15(1.77)	6(0.71)		-
Doctoral degree	0(0)	10(1.18)	7(0.83)		-
**Title (N(%))**				χ^2^(8) = 23.10^**^	
No professional title	9(1.06)	21(2.48)	24(2.83)		1 = 2 < 3
Junior professional title	107(12.63)	238(28.1)	103(12.16)		-
Intermediate professional title	76(8.97)	121(14.29)	46(5.43)		-
Associate senior professional title	30(3.54)	42(4.96)	14(1.65)		-
Senior professional title	5(0.59)	6(0.71)	5(0.59)		-
**Position(N(%))**				χ^2^(8) = 38.87^***^	
Medical position	95(11.22)	127(15)	44(5.2)		1 = 2 < 3
Nursing position	96(11.33)	192(22.67)	74(8.74)		1 = 2 < 3
Medical technology	28(3.31)	65(7.68)	43(5.08)		-
Laboratory testing	5(0.59)	25(2.95)	15(1.17)		-
Medical administration	3(0.35)	19(2.24)	16(1.90)		-
Working years (*M* ± *SD*)	10.20 ± 6.76	10.16 ± 8.02	9.69 ± 8.58	*F*(2,844) = 0.29, *η*^*2*^ = 0.001	-
Age (*M* ± *SD*)	34.69 ± 6.00	34.57 ± 7.18	33.91 ± 8.00	*F*(2,844) = 0.75, *η*^*2*^ = 0.002	-
**Variables (*M*** **±** ***SD*)**					
Work requirement	49.63 ± 10.63	43.31 ± 10.27	37.41 ± 10.48	*F*(2,844) = 72.15, *η*^2^ = 0.15, *p* < 0.001	1 > 2 > 3
Rumination	11.88 ± 2.36	10.52 ± 2.29	9.44 ± 1.85	*F*(2,844) = 64.59, *η*^2^ = 0.13, *p* < 0.001	1 > 2 > 3
WR	40.53 ± 4.77	54.97 ± 4.68	70.21 ± 5.22	*F*(2,844) = 1965.66, *η*^2^ = 0.82, *p* < 0.001	1 < 2 < 3
Mental distress	20.51 ± 10.34	11.92 ± 7.88	5.54 ± 6.27	*F*(2,844) = 173.87, *η*^2^ = 0.29, *p* < 0.001	1 > 2 > 3

Notes. *N* = 847; ^*^p < 0.05, ^**^p < 0.01, ^***^p < 0.001

Descriptive statistics and bivariate correlations for all variables are provided in the Table 3. WR showed positive correlations with fertility, marital status, and position, and significant negative correlations with work requirements, rumination and mental distress. These correlated factors were examined as predictors of WR classes.

**Table 3:** Descriptive statistics and correlations among variables

Variables	M ± SD	1	2	3	4	5	6	7
1. Fertility	-	-						
2. Marriage	-	0.65^**^	-					
3. Title	-	-0.27^**^	-0.21^**^	-				
4. Position	-	-0.02	0.001	-0.24^**^	-			
5. Work requirement	43.67 ± 11.26	-0.05	-0.06^*^	0.15^**^	-0.25^**^	-		
6. Rumination	10.64 ± 2.38	-0.03	-0.003	-0.001	-0.11^**^	0.36^**^	-	
7. WR	54.56 ± 11.48	0.09^**^	0.08^**^	-0.01^**^	0.14^**^	-0.42^**^	-0.40^**^	-
8. Mental distress	12.77 ± 9.85	-0.04	-0.06^*^	0.02	-0.11^**^	0.46^**^	0.53^**^	-0.61^**^

Note: *N* = 847. ^*^p < 0.05, ^**^p < 0.01, ^***^p < 0.001

Table 4 presents the results of multinomial logistic regression analysis using correlated factors as class predictors, with the high WR class as the reference. Age and gender were covariates. Position emerged as a potential risk factor for classification into the low WR class, with medical doctors more likely to fall into this category (OR = 5.08; 95% CI: 1.07–24.03). The low WR class reported higher work requirements (OR = 1.05; 95% CI: 1.02–1.07) and mental distress (OR = 1.24; 95% CI: 1.19–1.29) compared to the high WR class. The moderate WR class also showed higher levels of mental distress (OR = 1.14; 95% CI: 1.01–1.18) than the high class.

**Table 4:** Multinomial logistic regression results predicting latent class membership

Predictor	Low WR Class (50.5%)				Moderate WR Class (22.7%)				High WR Class (26.8%)
	M ± SD	*p*	OR	95% CI	M ± SD	*p*	OR	95% CI	M ± SD
Fertility (Ref: without children)									
With children	-	0.48	1.34	0.60–2.97	-	0.95	0.98	0.52–1.85	-
**Marriage** **(Ref: unmarried)**									
Married	-	0.21	1.80	0.72–4.54	-	0.28	1.48	0.73-3.00	-
widowed	-	-	**-**	**-**	-	0.88	0.81	0.05–14.56	-
Divorced	-	0.57	1.51	0.36–6.31	-	0.30	0.53	0.16–1.76	-
**Title** **(Ref: Senior professional title)**									
No professional title	-	0.77	1.36	0.40-12.45	-	0.97	0.97	0.21–4.60	-
Junior professional title	-	0.43	2.04	0.35–11.78	-	0.31	2.04	0.51–8.12	-
Intermediate professional title	-	0.25	2.78	0.49–15.92	-	0.21	2.42	0.61–9.61	-
Associate senior professional title	-	0.19	3.46	0.54–22.16	-	0.13	3.12	0.71–13.73	-
**Position** **(Ref: medical administration)**									
Medical position	-	**0.04**	5.08	1.07–24.03	-	0.47	1.41	0.55–3.58	-
Nursing position	-	0.09	3.63	0.81–16.19	-	0.42	1.43	0.60–3.38	-
Medical technology	-	0.18	2.92	0.61–13.96	-	0.92	0.96	0.39–2.38	-
Laboratory testing	-	0.39	2.25	0.36–14.06	-	0.47	1.48	0.51–4.28	-
**Work requirement**	49.63 ± 10.63	**< 0.001**	1.05	1.02–1.07	37.41 ± 10.48	0.08	1.02	1.00-1.04	43.31 ± 10.27
**Rumination**	11.88 ± 2.36	0.12	1.11	0.97–1.26	9.44 ± 1.85	0.54	1.04	0.93–1.15	10.52 ± 2.29
**Depression anxiety and stress**	20.51 ± 10.34	**< 0.001**	1.24	1.19–1.29	5.54 ± 6.27	**< 0.001**	1.14	1.10–1.18	11.92 ± 7.88

Note: The high WR class was the reference class; OR: odds ratio; ^*^p < 0.05, ^**^p < 0.01, ^***^p < 0.001

### Testing chain mediation effects based on WR classes

**Fig. 2:**
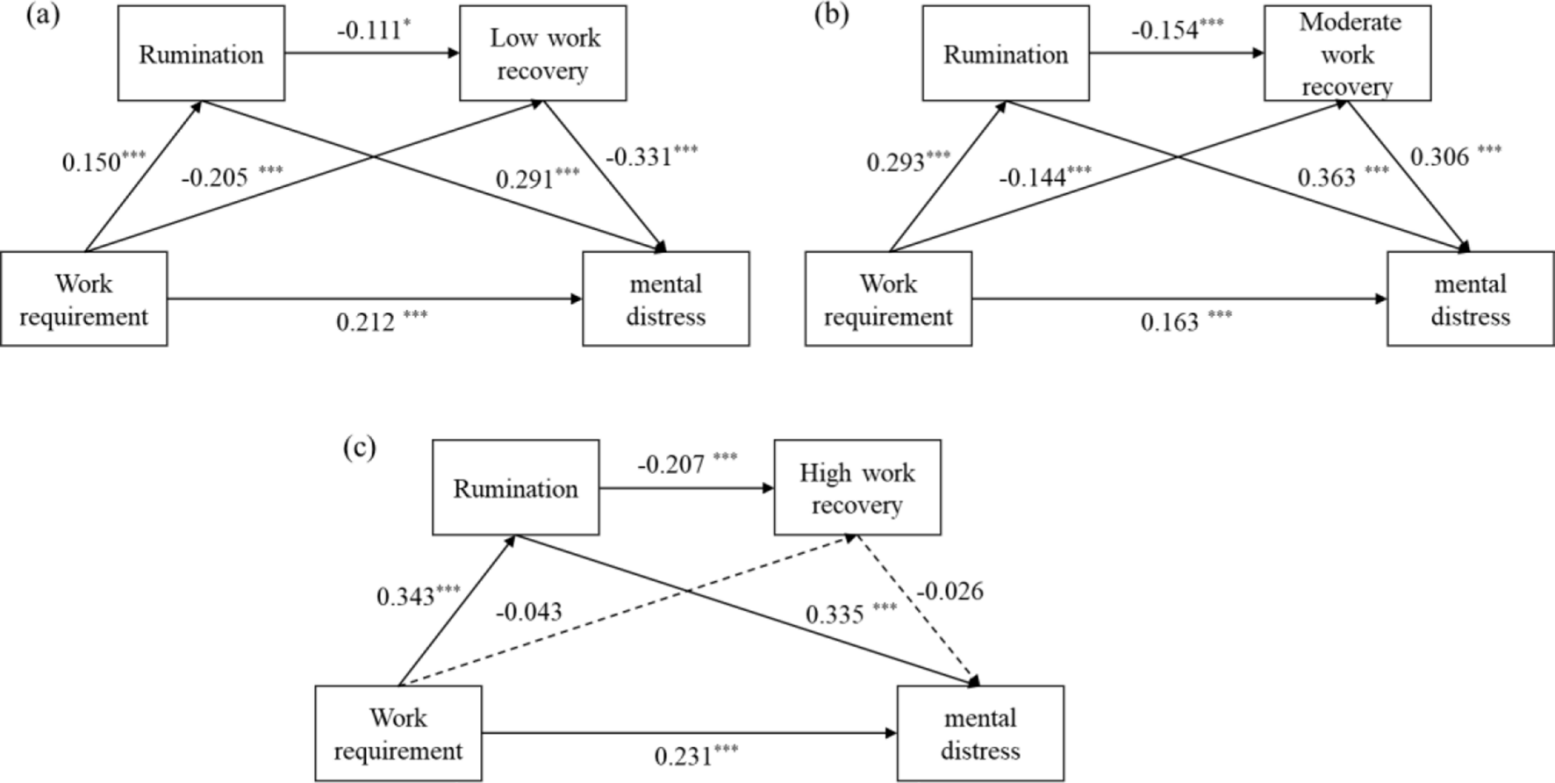
Chained Mediation Models Based on WR Classes

Figure 2 demonstrates the relationships among work requirements, mental distress, and rumination. This effect is significant in low and moderate WR classes but not in the high WR class. Rumination and WR classes mediate the relationship between work requirements and mental health outcomes, with mediation values of 0.134 and 0.143, respectively. The mediation consists of three indirect effects (Table 5): indirect effect 1 (low WR: 0.049; moderate WR: 0.093), indirect effect 2 (low WR: 0.075; moderate WR: 0.038), and indirect effect 3 (low WR: 0.010; moderate WR: 0.012). Bootstrap 95% confidence intervals for these effects do not include 0, indicating significance. In the high WR class, only indirect effect 1 (0.078) is significant. These findings suggest that staff with high WR are less affected by rumination under heavy work requirements, reducing their risk of mental distress. Conversely, those with low to moderate WR are more vulnerable to mental health issues due to higher rumination and lower WR. This also confirms the hypothesis proposed earlier, that rumination and WR will sequentially mediate the relationship between work requirements and mental distress for staff with impaired WR, but not for those with adequate WR.

**Table 5:** The results of the chain mediation effect values and bootstrap test for different WR classes

The Chain Mediation Effect	Effect Size	95% CI		Effect Size Ratio
		Lower Limit	Upper Limits	
The Chain Mediation Model of Low WR Class				
Work requirement → rumination →mental distress (Indirect Effect 1)	0.049^***^	0.007	0.097	0.132
Work requirement → low WR →mental distress (Indirect Effect 2)	0.075^***^	0.020	0.147	0.203
Work requirement → rumination → low WR →mental distress (Indirect Effect 3)	0.010^*^	0.001	0.018	0.027
Work requirement →mental distress (Direct Effect)	0.236^***^	0.111	0.361	0.638
**The Chain Mediation Model of Moderate WR Class**				
Work requirement → rumination →mental distress (Indirect Effect 1)	0.093^***^	0.057	0.133	0.325
Work requirement → moderate WR →mental distress (Indirect Effect 2)	0.038^***^	0.014	0.066	0.133
Work requirement → rumination → moderate WR →mental distress (Indirect Effect 3)	0.012^**^	0.004	0.022	0.042
Work requirement →mental distress (Direct Effect)	0.143^***^	0.072	0.214	0.500
**The Chain Mediation Model of High WR Class**				
Work requirement → rumination →mental distress (Indirect Effect 1)	0.078^***^	0.026	0.035	0.328
Work requirement → good WR →mental distress (Indirect Effect 2)	0.001	-0.009	0.009	0.004
Work requirement → rumination → good WR →mental distress (Indirect Effect 3)	0.001	-0.007	0.010	0.004
Work requirement →mental distress (Direct Effect)	0.158^***^	0.066	0.250	0.664

Notes. *N* = 847. ^*^p < 0.05, ^**^p < 0.01, ^***^p < 0.001

## Discussion

This study used LPA to determine the low, moderate, and high work recovery (WR) categories among hospital employees. Meanwhile, research has found significant differences in job requirements, rumination, WR, and mental distress among employees in different WR categories. Low and moderate WR employees report higher job requirements, more pronounced rumination, lower WR levels, and higher levels of mental distress. In addition, the impact of job requirements on mental distress, as well as the chain mediation effect of rumination and WR categories, is only significant among low and moderate WR employees. The impact mechanism of job requirements on mental health outcomes among high WR employees is different from that of low and moderate WR employees, and is less affected by rumination.

This study uses LPA to create three profiles representing the level of WR in Chinese hospital staff. They were low, moderate and high WR, which accounted for 26.8%, 50.5%, and 22.7%, respectively. The findings of this study differ from those of Sun et al. (2024) [41], who classified the recovery needs of construction workers into four distinct classes. This is probably because the nature of work, sources of stress, and work environments differ significantly between construction workers and hospital staff. Furthermore, Sun et al. (2024) use the variable of work recovery need which is different from the variable of WR in this study [41]. The differences in participant populations and the variables likely contribute to the variation in classifications. Our study underscores the importance of considering the heterogeneity in recovery across different occupations.

Our findings indicate that the low and moderate WR classes comprised the largest proportion of the sample, accounting for 77.3% of the hospital staff. The WR scores for the low and moderate classes were about 40 and 54, a large gap from the scores of the high WR class (higher than 70), showing a giant bipartition trend. In addition, there were significant differences between classes in variables related to WR, especially depression anxiety and stress, which represent the level of mental health. The scores of depression anxiety and stress in the low and moderate WR classes were 20 and 11, which showed a significant gap with the scores of the high WR class (lower than 6), indicating a bipartition trend. This result indicates that hospital staff in low and moderate WR class may have more recovery and mental health issues compared with high WR class. Therefore, it is essential to develop different treatment options based on classes to improve the WR and decrease depression anxiety and stress among hospital staff. This study used latent profile analysis (LPA) to classify the WR of hospital employees into three categories: low, moderate, and high. Based on this, different intervention suggestions were proposed for hospital employees in different WR categories: for employees in the low WR category, they scored the lowest in all WR dimensions, had higher job requirements, rumination levels, and mental distress levels. It is recommended to provide them with specialized psychological counseling services, such as regularly organizing one-on-one psychological counseling to help them identify sources of work stress, change negative thinking patterns, and reduce rumination. Considering their poor work recovery ability, simple and easy relaxation training can be introduced, such as deep breathing exercises during work breaks, brief meditation training, etc., to help them quickly relieve work pressure and gradually improve their work recovery ability. The indicators of employees in the moderate WR category are at an intermediate level, but their mental distress level is still relatively high. Therefore, cognitive-behavioral therapy (CBT) group courses can be conducted to help them identify and adjust negative thinking and behavior patterns that cause stress and hinder recovery. Encourage them to develop reasonable work plans and rest schedules, ensure a balance between work and recovery, improve work recovery efficiency, and reduce levels of psychological symptoms. The overall psychological state of employees in the high WR category is good, but it also needs to be maintained and further improved. We can provide guidance on career development planning to help them achieve their career goals on the basis of a good job recovery, further enhancing their sense of achievement and satisfaction in their work. Experience sharing sessions can also be organized for employees in high WR categories to share their work recovery experiences and skills, promoting communication and learning among different categories of employees.

This study examined the chain mediating effects of rumination and work recovery (WR) classes on the relationship between work requirements and symptoms of depression, anxiety, and stress. The findings indicate that both moderate and low WR classes mediate the association between work requirements and these psychological symptoms. Specifically, hospital staff in moderate and low WR classes reported higher perceptions of work requirements and were more likely to engage in ruminative thoughts and experience impaired recovery, leading to increased depressive, anxious, and stressful symptoms compared to those in the high WR class. These results highlight that hospital staff with low and moderate WR are at a heightened risk for adverse health outcomes, aligning with existing literature that suggests rumination and impaired recovery contribute to detrimental psychological states [23, 53]. Long-term accumulation of impaired WR has been shown to have a more profound impact on both physical and psychological health compared to rumination [23, 54]. Therefore, it’s important to note that impaired WR is an important pathway from work requirements to psychological health problems, indicating that it can be a severe precursor to depression, anxiety and other mood disorders. Although hospital staff have the highest depressive, anxious, and stressful rates among many stressful occupations, the proportion of the population accessing healthcare services for depression and anxiety disorders is deficient compared to other occupations [55–57]. This is reflective of negative social perceptions surrounding depression, anxiety and other mood disorders in hospital staff [55, 58]. There is a tendency to avoid psychiatric treatment or counselling for depression in hospital staff due to the misconception that receiving psychiatric treatment will cause social disadvantages in areas such as employment, marriage, or insurance [59, 60]. These circumstances make it difficult to detect and intervene early in high-risk hospital staff for depression, anxiety, and other mood disorders. Thus, policymakers should pay more attention to the development of interventions targeting WR may enable high-risk health medical staff to eliminate their bias towards psychological intervention and thus better accept intervention.

According to our findings, future research can help provide hospital staff with interventions for improving WR. There are relatively few interventions that specifically address how to improve the WR of hospital staff, future research might focus on exploring the most effective interventions. There may be some interventions to enhance WR: (1) Mindfulness-Based Interventions: Practices such as mindfulness meditation and yoga can help reduce stress and enhance emotional regulation, leading to improved WR [61]. (2) Resilience Training: Programs designed to build resilience skills can help medical staff cope with stressors and recover more effectively from work-related fatigue [62]. (3) Cognitive Behavioral Therapy (CBT): CBT can assist individuals in identifying and modifying negative thought patterns that contribute to stress and hinder recovery [63]. However, these psychological interventions typically need to be carried out over a more extended period and by qualified psychologists. So, most medical staff are knowledgeable about health issues, and the intense work requirements and lack of personal time make it difficult for them to undertake these complex interventions. In addition, the majority of the interventions focus on psychological issues like anxiety and depression and how to control them, which may not be chosen by medical staff due to the stigma attached to depression and anxiety. Therefore, future research can focus on the development of simple intervention methods that do not require the participation of qualified psychologists to improve WR. In the current medical work environment, medical staff have high work intensity and limited personal time, and traditional intervention methods that require the deep involvement of professional psychologists are often difficult to implement. Therefore, it is urgent to develop more convenient and easy-to-use intervention measures. Although some online or application interventions are currently ineffective [64], this does not mean that all such interventions are not feasible. We can improve and explore from the following aspects: (1) Personalized intervention design. By utilizing big data and artificial intelligence technology, tailored intervention plans are developed for healthcare workers based on their different types of work recovery, rumination levels, and mental health conditions. For example, for medical staff with low work recovery and high rumination, specialized relaxation training, cognitive restructuring exercises, and other content can be pushed to improve the targeted and effective intervention. (2) Gamification and interactive experience. Design the intervention content as a game or interactive module to increase fun and engagement. By setting up checkpoints, reward mechanisms, etc., encourage medical staff to actively participate in the intervention process and improve their compliance. For example, developing a mobile game with the theme of relieving work stress, where players complete various simulated work scenarios to learn and apply effective psychological adjustment strategies. (3) Peer support and mutual assistance community: Establish an online mutual assistance community among medical staff, allowing them to share their work pressure, experience, and coping strategies. Mutual understanding and support among peers are often more approachable and persuasive, and this social interaction itself can also help alleviate psychological stress. Communities can be equipped with administrators who have undergone simple training, responsible for guiding discussion directions and providing basic psychological support knowledge, but professional psychologists do not need to be involved throughout the entire process. (4) Combining wearable devices: By using wearable devices such as smart bracelets and smartwatches to collect physiological data (such as heart rate, sleep quality, etc.) of medical staff, combined with their psychological state data, their physical and mental health status can be monitored in real time. Based on this data, timely personalized intervention recommendations should be pushed to medical staff, such as reminders for relaxation exercises such as deep breathing and meditation when abnormal heart rate elevation is detected.

However, this study has several limitations: (1) Sample Representativeness: Although medical staff were selected as participants, the diversity within this group may limit the generalizability of the research findings. Future studies should encompass a broader range of medical staff demographics to enhance the applicability of the results across different healthcare settings. (2) Cross-Sectional Design Constraints: The cross-sectional design of this study may restrict a comprehensive understanding of causal relationships. Future research should employ longitudinal study designs to better elucidate the causal relationships between work requirements, rumination, WR classes, and depression anxiety and stress. (3) Diversity in WR: While this study primarily focuses on psychological WR, it is essential to consider physiological indicators related to WR, such as cortisol and heart rate variability [65]. Subsequent research should investigate the impact of these physiological indicators, thereby deepening our understanding of the roles that different WR classes play in the medical staff.

## Conclusion

This study identified low, moderate and high WR classes among hospital staff. There were significant associations between work requirements and mental distress mainly among medical staff in the low and moderate WR classes. Furthermore, the chain mediation effects of rumination and WR classes were also only significant within these two classes.

## Electronic supplementary material

Below is the link to the electronic supplementary material.

Supplementary Material 1

## Data Availability

No datasets were generated or analysed during the current study.

## Abbreviations

WR: Work Recovery
LPA: Latent Profile Analysis
WRS: Work Requirements Scale
RESTQ: Recovery - Stress Questionnaire
DASS: Depression Anxiety Stress Scales
RECQ: Revised Emotion Control Questionnaire
