# Supplementary material for: The relationship between work requirements and mental distress in hospital staff: the chain mediating effects of rumination and work recovery classes
Source: BMC Psychol. 2025 Mar 17;13:256. doi: 10.1186/s40359-025-02588-1 (PMC11912755; doi:10.1186/s40359-025-02588-1)
Supplement: Supplementary file 1 — Supplementary Material 1 [file 40359_2025_2588_MOESM1_ESM.pdf]

附件 9：

贵州中医药大学第二附属医院医学伦理委员会审批函

项目名称	医护人员身心健康研究				
伦理批准号	EC202003		项目起止时间	2023 年 3 月 21 日-2024 年 3 月 21 日	
项目类别	☐药物临床试验 ☐医疗器械临床试验<br>√科研 √调查 ☐流行病学 ☐数据采集 ☐遗传研究<br>☐干预 ☐样本采集 ☐动物实验 ☐其他				
主要研究者姓名	肖政华	学历/学位	博士	联系电话	18984040248
				邮箱	xiaozhenghua@126.com
研究内容	本研究旨在全面调查医护人员的身心健康状况,涵盖工作压力、睡眠质量、心理健康水平及其相关影响因素。医护人员长期处于高强度、快节奏的工作环境中,面临着较大的心理和生理负担,这不仅影响其个人健康,也可能间接影响医疗服务质量,因此,本研究希望通过系统收集和分析相关数据,识别主要风险因素,揭示身心健康状况与工作环境之间的关系,为制定更科学、有效的健康管理策略和工作支持措施提供实证依据,从而提升医护人员的福祉,并进一步优化医疗体系的可持续发展。				

项目申请人（或主要研究者）承诺：

以上填写内容属实，若获批准，本人＿＿肖政华＿＿将严格按照提供的方案进行研究，遵守医院伦理委员会的相关规定，接收伦理委员会的监督，

日期： 2023 年 2 月 20 日

医院伦理委员会审批意见：

经审查本研究项目，研究者资格符合研究要求，研究设计和方法合理，有风险预防和应对措施，符合伦理学审查的各项原则， 同意开展此项研究。

主任委员签章：张敬杰印

贵州中医药大学第二附属医院医学伦理委员会

2023 年 3 月 21 日

本次备案材料见附件，

版本号：GZDEFY-LSPJ1.0
